# Seasonal and Extraction-Dependent Variation in the Composition and Bioactivity of Essential Oils from Wild *Rosmarinus officinalis* L.

**DOI:** 10.3390/molecules30214258

**Published:** 2025-10-31

**Authors:** Khalil Guelifet, Khaled Kherraz, Mohammed Messaoudi, Mohamed Amine Ferhat, Latifa Khattabi, Khadra Afaf Bendrihem, Wafa Zahnit, Dalila Addad, Mokhtar Benmohamed, Yacine Azoudj, Lilya Harchaoui, Khaled Aggoun, Abdenour Boumechhour, Luca Rastrelli

**Affiliations:** 1 Laboratoire de Recherche sur les Produits Bioactifs et Valorisation de la Biomasse, Département de Chimie, ENS Vieux-Kouba Alger, Algiers 16050, Algeria; khalil.guelifet@g.ens-kouba.dz (K.G.); ferhatamine100@yahoo.fr (M.A.F.); 2 Laboratoire d’Ethnobotanique et Substances Naturelles, Ecole Normale Supérieure, Vieux-Kouba Algiers, Algiers 16050, Algeria; khaled.kherraz@g.ens-kouba.dz; 3 Biotechnology Research Center (CRBt), Constantine 25016, Algeria; l.khattabi@crbt.dz; 4 Biotechnology, Water, Environment and Health Laboratory, Faculty of Natural and Life Sciences, University of Abbes Laghrour, Khenchela 40000, Algeria; afaf.bendrihem5@gmail.com (K.A.B.); dalilaaddad@gmail.com (D.A.); 5 Department of Chemistry, Faculty of Sciences, University of Ferhat ABBAS Setif 1, El Bez 19000, Algeria; zahnit_07_hanane@outlook.fr; 6 Laboratory of Fundamental Sciences, University Amar Télidji of Laghouat, P.O. Box. 37G, Road of Ghardaïa, Laghouat 03000, Algeria; msbm1447@gmail.com; 7 Centre de Recherche Scientifique et Technique en Analyse Physico-Chimiques (CRAPC), Zone Industrielle Bou-Ismail RP, P.O. Box. 384, Tipazan 42004, Algeria; yacine.a2h@hotmail.com; 8 Faculty of Biological Sciences, University of Science and Technology Houari Boumediene, Algiers 16111, Algeria; lilya.h@live.fr; 9 Laboratory of Organic Materials and Heterochemistry, Echahid Cheikh Larbi Tebessi University, Constantine Road, Tebessa 12002, Algeria; khaled.aggoun@univ-tebessa.dz; 10 Centre for Scientific and Technical Research in Analysis Physico-Chemicals (CRAPC), Algiers 16111, Algeria; 11 Department of Pharmacy, University of Salerno, Via Giovanni Paolo II, 132, 84084 Fisciano, Italy; 12 National Biodiversity Future Center (NBFC), 90133 Palermo, Italy

**Keywords:** *Rosmarinus officinalis* L., essential oils, harvest season, chemical composition, biological properties, seasonal variability, GC-MS analysis, extraction methods

## Abstract

This study investigated the impact of harvest season and extraction method on the yield, composition, and bioactivity of essential oils (EOs) from wild *Rosmarinus officinalis* L. plants collected in Algeria. Oils were obtained by hydro distillation (HD), steam distillation (SD), and microwave-assisted distillation (MD) across four seasons and characterized by GC–MS. Camphor, α-pinene, camphene, and 1,8-cineole were consistently dominant, with spring oils, particularly those extracted by microwave-assisted distillation, showing the highest enrichment in oxygenated monoterpenes (up to 59.6%). Functional assays revealed clear seasonal variation, whereas spring oils exhibited the strongest antioxidant capacity, with a FRAP value of 4.63 µg/mL, approaching that of the synthetic standard BHA (6.89 µg/mL), alongside notable anti-inflammatory effects. Antimicrobial screening indicated selective inhibition of *Escherichia coli* and *Candida albicans*, while *Pseudomonas aeruginosa* and *Bacillus subtilis* remained resistant. Acute toxicity evaluation confirmed safety at 2000 mg/kg. These findings demonstrate that ecological timing and extraction strategy critically determine rosemary EO properties and establish quantitative benchmarks for their pharmaceutical and industrial valorization.

## 1. Introduction

*Rosmarinus officinalis* L. (syn. *Salvia rosmarinus* Spenn.), commonly known as rosemary, is an aromatic member of the Lamiaceae family native to the Mediterranean region. Its essential oil (EO), rich in volatile monoterpenes such as 1,8-cineole, camphor, and α-pinene, is widely used in culinary, pharmaceutical, and cosmetic products due to its antioxidant, antimicrobial, and anti-inflammatory properties [1,2]. Beyond traditional uses, rosemary EO has gained industrial importance as a natural preservative and functional ingredient [3].

Despite its broad application, the composition and activity of rosemary EO are highly variable, shaped by both environmental and technological factors. Seasonal fluctuations in temperature, rainfall, and light exposure influence the biosynthesis of volatile metabolites, while extraction method strongly determines oil yield and chemical profile [4].

Conventional hydro distillation (HD) and steam distillation (SD) remain standard, but microwave-assisted distillation (MD) has emerged as a promising alternative, improving recovery efficiency and preserving oxygenated compounds sensitive to heat degradation [5].

While individual studies have addressed either seasonal variation or extraction technique, comprehensive comparative assessments integrating both factors across complete annual cycles remain scarce, particularly for wild North African populations. Moreover, conflicting reports on composition–bioactivity relationships, such as the paradox between higher phenolic content in autumn and stronger antioxidant activity in spring, warrant systematic re-evaluation [6,7,8,9].

This study investigates the influence of harvest season and extraction method (HD, SD, MD) on yield, composition, and biological activities of wild Algerian rosemary oils. GC–MS was used to characterize chemical profiles, while antioxidant, antimicrobial, and anti-inflammatory assays evaluated functional properties.

The findings aim to identify optimal harvest–extraction combinations for producing high-quality rosemary EO with consistent bioactivity, potentially reducing batch-to-batch variation in commercial formulations and supporting evidence-based standardization protocols.

## 2. Results and Discussion

### 2.1. Effect of Extraction Method on the Yield

Essential oils from *Rosmarinus officinalis* were obtained using three different techniques: hydro distillation (HD), microwave-assisted distillation (MD), and steam distillation (SD). Samples were collected across four seasons to evaluate yield variations. The results (Table 1) indicate that HD produced the highest yields, whereas MD consistently resulted in the lowest. The maximum yield (0.81%) was achieved in spring using HD, while the minimum (0.25%) was obtained in winter with MD. Previous studies have similarly reported yield variations depending on plant variety and environmental conditions. Bousbia et al. [10] noted differences between cultivated and wild rosemary and across extraction techniques. For instance, in semi-humid Algerian regions (Algiers and El Kala), fresh and dry aerial parts harvested in March and April yielded 0.82% and 0.36%, respectively, when extracted by HD. In the present study, SD yielded consistently lower amounts than HD but higher than MD across all seasons. The percentage of EO obtained with SD over four seasons ranged between 32% and 78% relative to HD, which is consistent with Mateus et al. [11], who reported yields of 0.5–1.5%, and Arafa [12], who found values between 0.77% and 1.03%. By contrast, MD produced the lowest yields (25–65%), aligning with Elyemni et al. [13], who reported MD yields of 32–39%.

### 2.2. Effect of Extraction Method on the Chemical Composition of Essential Oil

Gas chromatography–mass spectrometry analysis of *Rosmarinus officinalis* essential oils extracted across four seasons using hydro distillation, steam distillation, and microwave-assisted distillation identified 47 compounds, accounting for 87.7–99.0% of total composition (Table 1). The oils were dominated by monoterpene hydrocarbons (28.8–50.14%) and oxygenated monoterpenes (38.2–59.65%), with lower proportions of sesquiterpene hydrocarbons (2.29–12.14%) and oxygenated sesquiterpenes (0.92–5.44%). These profiles are consistent with Mediterranean rosemary chemotypes [14], though the quantitative distribution reflects both seasonal regulation and method-specific retention.

Camphor consistently emerged as the predominant compound across all seasons and extraction methods, with concentrations ranging from 21.95% to 36.52%. This was followed by α-pinene (7.31–21.72%), camphene (7.84–18.23%), and 1,8-cineole (7.65–14.53%). Other relevant constituents included borneol (1.42–6.57%), 4-terpineol (0.82–6.48%), and α-terpineol (0.58–2.72%). These findings clearly establish our wild rosemary population as belonging to the camphor–α-pinene chemotype, distinguishing it from the verbenone–bornyl acetate chemotype common in cultivated varieties and the 1,8-cineole–camphor chemotype prevalent in some Mediterranean regions. This camphor–α-pinene chemotype is consistent with the Romanian type but distinct from the Tunisian reference (52.77% 1,8-cineole) [15].

**Figure 1:**
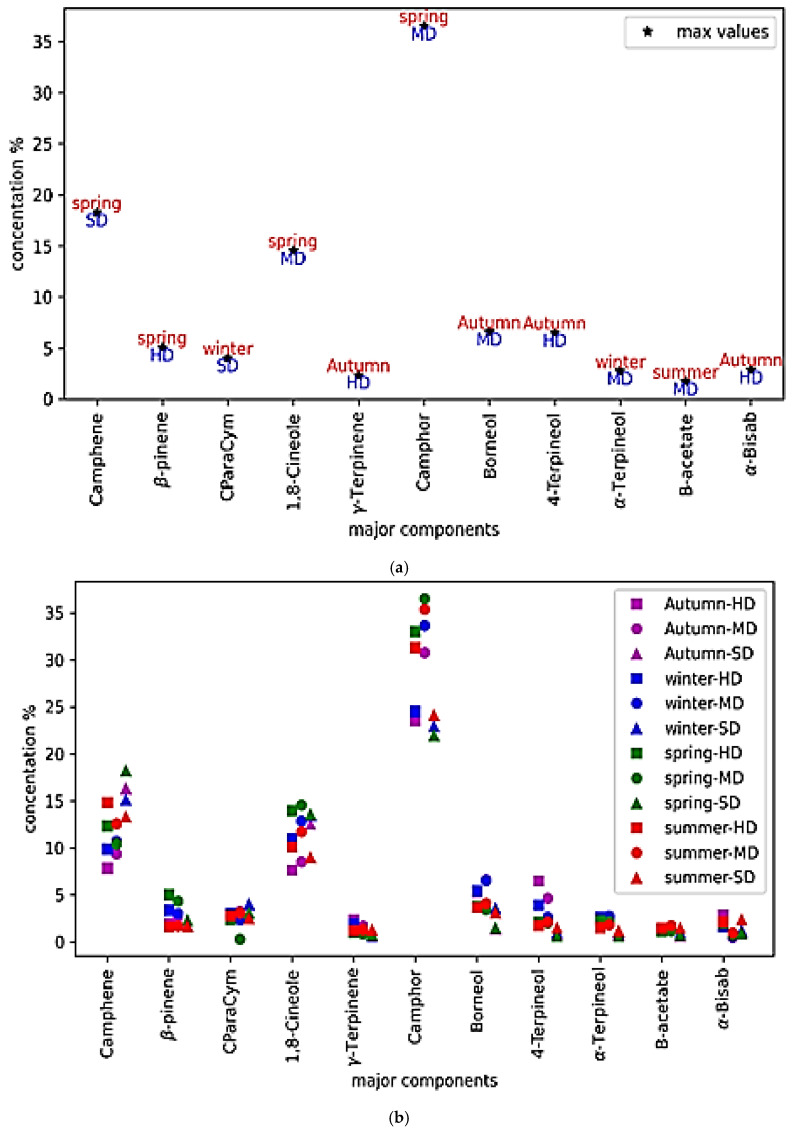

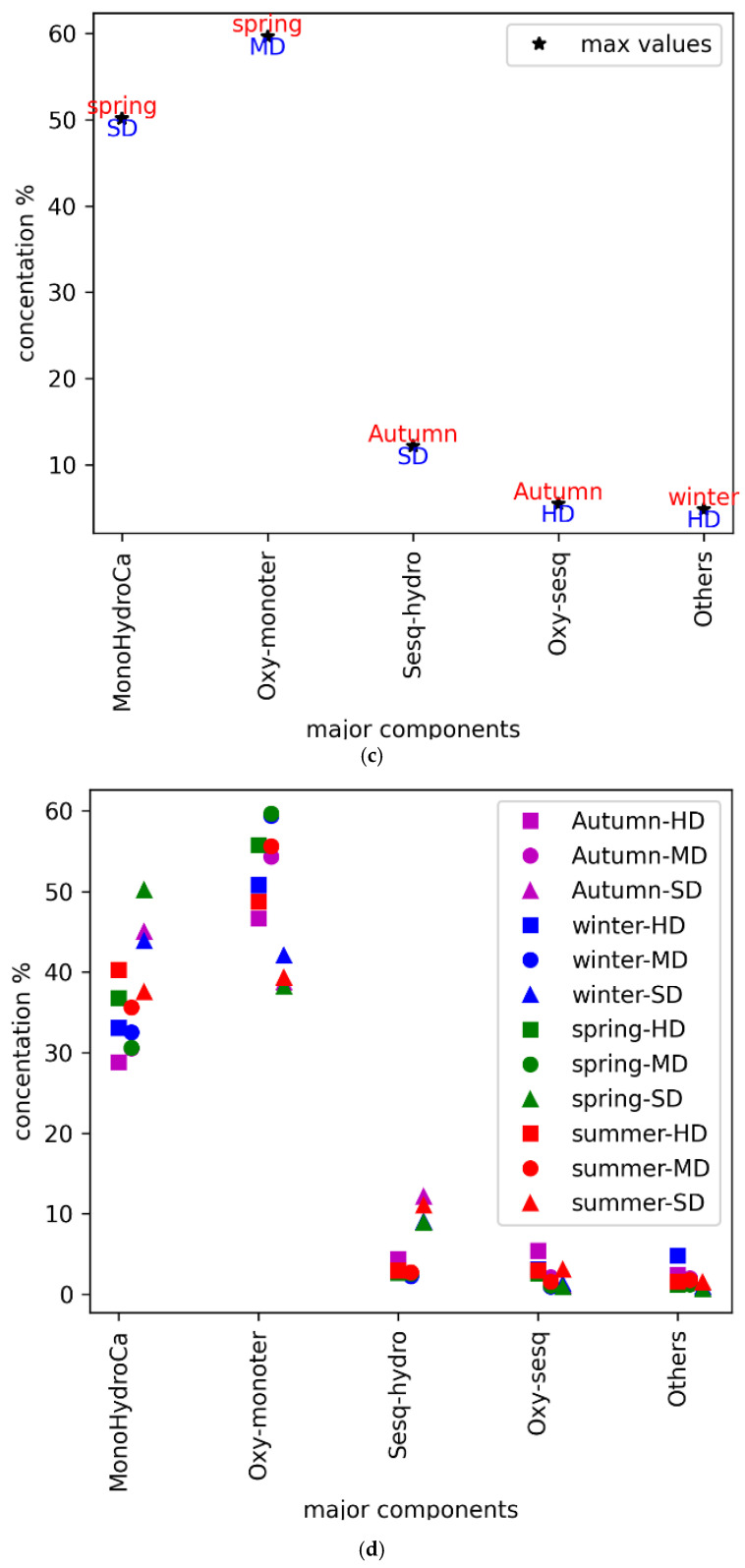
Seasonal and methodological variation in the chemical composition of *Rosmarinus officinalis* essential oils. (**a**) Relative concentrations (%) of main volatile constituents identified by GC–MS across seasons and extraction methods, highlighting maximum values. (**b**) Comparison of major constituents (α-pinene, camphene, 1,8-cineole, camphor, borneol, and others) obtained by hydrodistillation (HD), steam distillation (SD), and microwave-assisted distillation (MD) in autumn, winter, spring, and summer. (**c**) Distribution of chemical classes (monoterpene hydrocarbons, oxygenated monoterpenes, sesquiterpene hydrocarbons, oxygenated sesquiterpenes, and others) as a function of extraction method. (**d**) Seasonal and methodological clustering of major chemical groups, showing the predominance of oxygenated monoterpenes in microwave-distilled spring oils.

Marked seasonal and methodological differences were evident across all major constituents (Table 1). Spring harvests consistently yielded the highest proportion of identified compounds (98.92–99.02%), enriched particularly in monoterpene hydrocarbons such as α-pinene (up to 21.72% with steam distillation) and camphene (18.23%), together with elevated camphor (36.52% with microwave distillation) and 1,8-cineole (14.53%). These profiles are consistent with enhanced volatile biosynthesis during the flowering period (Figure 1) and with upregulation of the methylerythritol phosphate pathway reported in other aromatic Lamiaceae species [15]. The elevated spring monoterpene content reflects increased metabolic activity during reproduction, as these volatile compounds serve both defensive and pollinator-attracting functions [16]. Such seasonal trends are consistent with the observations of Lakušić et al. [17], who demonstrated that environmental and phenological factors can modulate chemotype expression even within the same rosemary genotype.

In contrast, autumn and summer oils contained substantially higher proportions of sesquiterpene hydrocarbons (autumn: 12.14% SD; summer: 11.07% HD) and oxygenated sesquiterpenes (autumn: 5.44% HD), including δ-cadinene, trans-caryophyllene, and caryophyllene oxide. These compounds are hypothesized to serve adaptive roles under elevated temperatures and water limitation typical of Mediterranean summers [16,18], though direct measurements of plant stress markers were not conducted in this study. Daussy and Staudt [18] demonstrated increased sesquiterpene emissions under thermal stress in Mediterranean aromatic species, supporting the hypothesis that heat enhances sesquiterpene biosynthetic enzyme activity. Comparable results were reported by Ben Arfa et al. [19] for Tunisian rosemary, where sesquiterpene contents were markedly lower (1.69–2.67%). Bejenaru et al. [20] likewise described strong seasonal shifts in Mediterranean rosemary, reinforcing the combined influence of genetics and environment on this compound class. Winter samples generally showed reduced metabolic activity across most compound classes, though several rare constituents—β-elemene, eugenol, cuminic aldehyde, and methyl eugenol—appeared exclusively in hydro distilled winter extracts, while trans-β-ocimene was confined to spring oils, reflecting the tightly regulated seasonality of specific biosynthetic pathways.

The extraction technique significantly modulated oil profiles. Microwave-assisted distillation consistently produced oils richest in oxygenated monoterpenes (54.27–59.65%), particularly camphor, 1,8-cineole, and borneol, with maximum concentrations observed in spring. The efficiency of this method can be attributed to rapid and uniform heating, which minimizes thermal degradation and preserves oxygenated volatiles [13].

Steam distillation yielded higher monoterpene hydrocarbons (45.02–50.14%) and sesquiterpene hydrocarbons (8.95–12.14%), while microwave-assisted distillation produced oils richer in oxygenated monoterpenes (54.27–59.65%), with camphor reaching 36.52% versus 28.47% in steam extracts. These differences reflect method-specific extraction selectivity based on compound volatility and heating mechanisms [21].

The chemotypic profile of Algerian rosemary reveals both convergence and divergence with global populations (Table 2). Camphor-dominant chemotypes are common in wild North African and cultivated South American rosemary, with our maximum camphor concentration (36.52 ± 1.8%) overlapping with reported values from Mexico (39.46%) [22] and Brazil (33.2%) [3], though formal statistical comparison is precluded by differences in sampling and analytical protocols. However, intra-regional variation is substantial: oils from the Algerian Beban region contained approximately half the camphor levels reported here (12.6–19.6%) [6], underscoring significant genetic or environmental influences even within Algeria. In contrast, Tunisian (52.77% 1,8-cineole) [15] and Moroccan (31.2% 1,8-cineole) [13] populations are dominated by 1,8-cineole rather than camphor, while Italian rosemary exhibits much lower camphor (4.9–11.3%) [23] and Sardinian populations maintain remarkably stable α-pinene content (26–28%) year-round [23], contrasting with our pronounced seasonal variation (7.31–21.72%). Himalayan rosemary maintains a 1,8-cineole–rich chemotype with minimal sesquiterpenes throughout the year [24], consistent with adaptation to high-altitude, monsoon-influenced environments fundamentally different from Mediterranean climates. These geographic differences reflect the interaction of genetic differentiation among wild and cultivated populations, environmental factors (temperature, water availability, soil composition, photoperiod), and phenological variation, though harvest timing is often unreported in the literature. Such diversity highlights the interplay of genotype, environment, and developmental stage in shaping chemotypic expression and complicates efforts toward industrial standardization.

### 2.3. Antioxidant Activity

**Figure 2:**
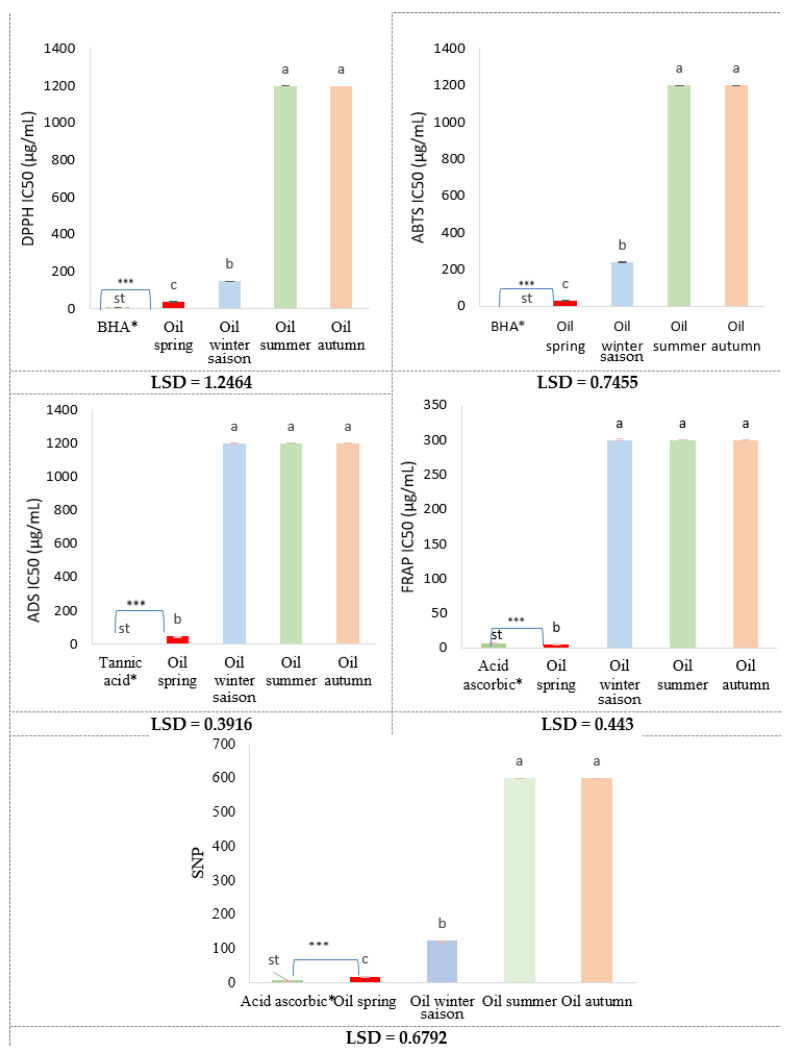
Seasonal variation in antioxidant activity of rosemary essential oils across all assays, with LSD groupings. Bars represent mean ± SD (n = 3). Different letters indicate significant differences among means according to the LSD test (*p* < 0.05). ***: The values are highly significant. *: Standard compounds.

The antioxidant potential of *Rosmarinus officinalis* essential oils (EOs) was evaluated using a panel of complementary in vitro assays. The results, summarized in Table 3 and Figure 2, revealed marked seasonal variation in antioxidant activity.

Among all samples, the spring oil (hydrodistillation) exhibited the strongest antioxidant capacity, with IC_50_ values of 39.22 µg/mL (DPPH), 30.71 µg/mL (ABTS), and 45.58 µg/mL (ADS), as well as A_0.5_ values of 4.63 µg/mL (FRAP) and 19.06 µg/mL (SNP). This consistent performance across assays correlates with its favorable chemical profile, enriched in monoterpene hydrocarbons (50.14%) and oxygenated monoterpenes (38.2%), including α-pinene (21.72%), camphene (18.23%), 1,8-cineole (13.59%), and camphor (21.95%). These compounds are known contributors to antioxidant activity through diverse mechanisms [31]. The observed activity is attributed to complementary antioxidant mechanisms. α-Pinene and camphene donate hydrogen atoms to neutralize peroxyl and alkoxyl radicals [32], explaining the strong DPPH and ABTS performance. 1,8-Cineole enhances antioxidant enzyme activity (e.g., SOD), stabilizes radicals, and activates Nrf2-dependent pathways [33,34,35,36], contributing to superoxide scavenging in the ADS assay. Camphor, despite its limited direct activity, acts synergistically with phenolic terpenes by stabilizing radical intermediates [34].

By contrast, the closely related species *Rosmarinus officinalis* L., collected in April, showed negligible antioxidant activity (IC_50_ > 200 µg/mL across assays), despite high 1,8-cineole levels (55.26%) [37]. This contrast highlights the importance of chemotypic composition and terpene synergy, as camphene, α-pinene, and borneol were more abundant in *R. officinalis*. In FRAP assays, spring oil displayed strong reducing power (A_0.5_ = 4.63 µg/mL), surpassing ascorbic acid (A_0.5_ = 6.77 µg/mL). This reflects high electron-donating capacity under acidic, iron-rich conditions, attributed to α-pinene and camphene [38], complemented by oxygenated monoterpenes such as 1,8-cineole, borneol, and α-terpineol [39]. Similar results were reported by [40], who showed that oxygenated terpenes display antioxidant activity comparable to phenolics. Beretta et al. [41] also demonstrated that antioxidant efficacy is shaped by the balance of key terpenes, rather than dominance of a single constituent. The SNP assay confirmed this trend, with spring oil showing strong reductive ability (A_0.5_ = 19.06 µg/mL), consistent with studies on plant-mediated silver nanoparticle synthesis [42]. Monoterpenes such as borneol and α-terpineol not only reduce silver ions but also stabilize nanoparticles by surface adsorption [43,44], reinforcing the technological potential of rosemary oil in nanomedicine and pharmaceuticals. Interestingly, Ref. [20] reported maximum antioxidant activity in Romanian *R. officinalis* during summer (IC_50_ = 2.69–3.92 µg/mL), associated with higher oxygenated monoterpenes (56.82–66.94%), particularly camphor (40.03%). By contrast, our spring oil, with a more balanced profile of hydrocarbons and oxygenated terpenes, excelled in electron transfer assays (FRAP, SNP). These results suggest that broad-spectrum antioxidant activity depends on compositional balance rather than dominance of a single compound. Similar findings were observed by Ben Arfa et al. [19], who linked peak antioxidant activity in Tunisian rosemary to flowering and early summer, correlating with higher camphor, camphene, and borneol levels. Seasonal influences were also confirmed by [37], who reported highest antioxidant and phenolic levels in winter and spring across various aromatic species. By contrast, summer and autumn oils showed weak antioxidant activity (IC_50_ > 1200 µg/mL; A_0.5_ > 300 µg/mL). Their reduced efficacy corresponds to lower antioxidant-related monoterpenes and enrichment in sesquiterpenes (δ-cadinene, trans-caryophyllene, α-humulene), which possess anti-inflammatory but limited radical-scavenging activity [45]. Their higher molecular weight and steric bulk reduce accessibility to radical sites [33]. However, sesquiterpenes may contribute indirectly by modulating cellular redox pathways [45]. The winter oil showed intermediate activity (DPPH IC_50_ = 148.96 µg/mL; ABTS IC_50_ = 240.54 µg/mL), consistent with its high camphor (33.63%) and 1,8-cineole (12.84%) content but a lack of synergistic balance seen in spring oils. This confirms that antioxidant efficacy is shaped by constituent interplay rather than dominance of single compounds. ANOVA results confirmed significant seasonal effects (*p* < 0.0001) across all assays. As illustrated in Figure 2, the seasonal variation in antioxidant activity is clearly visualized across all assays, with distinct groupings confirmed by the LSD post hoc test. Post hoc LSD comparisons (*p* < 0.05) revealed that spring oils exhibited significantly higher radical scavenging capacity than those collected in other seasons (LSD values: DPPH = 0.7455, ABTS = 1.2464, ADS = 0.443, FRAP = 0.3916, SNP = 0.6792).

### 2.4. Antimicrobial Susceptibility Assay In Vitro

The antimicrobial activity of *Rosmarinus officinalis* essential oils (EOs) extracted across the four seasons (autumn, winter, spring and summer) was evaluated against a panel of microbial strains, with Gentamicin (GNT) used as the reference antibiotic. Oils were tested at concentrations of 50%, 25%, 15% and 10% (Table 4).

The results revealed pronounced seasonal and dose-dependent variation in antimicrobial effects. The strongest antibacterial activity was observed against Escherichia coli, particularly in autumn, winter, and summer. At 50% concentration, inhibition zones reached 27 mm, comparable to Gentamicin (26–27 mm), suggesting that EOs harvested in these seasons contain potent antibacterial constituents. In contrast, spring oils exhibited markedly reduced activity (7–9 mm), highlighting the influence of seasonal chemical variation.

No inhibitory activity was observed against *Pseudomonas aeruginosa* or *Bacillus subtilis* in any season, suggesting either an inherent resistance of these microorganisms or a lack of strong antimicrobial compounds in rosemary oil. The resistance of *P. aeruginosa* aligns with its known low outer membrane permeability and the presence of constitutive efflux pump mechanisms, which actively remove lipophilic terpenoids before they can accumulate to inhibitory concentrations within the cell [46]. The well-documented resilience of *Bacillus* species, particularly their spore-forming ability and robust cell envelope structure, may also contribute to their low susceptibility to essential oils. Spores of *Bacillus subtilis* are known to resist heat, chemicals, and common biocides more effectively than vegetative cells [47,48]. Previous work has shown that many essential oils require high concentrations or prolonged exposure to exert sporicidal or inhibitory effects [49]. Furthermore, the correlation analysis revealed that compounds showing strong antimicrobial activity against *E. coli* did not exhibit correlations with activity against *P. aeruginosa* or *B. subtilis*, suggesting that these organisms require different chemical classes, higher concentrations of specific constituents, or synergistic combinations not present at sufficient levels in *R. officinalis* essential oil. This finding reinforces the organism-specific nature of essential oil activity. It underscores that the compositional profiles identified through PCA, which effectively discriminated oils based on *E. coli* and *C. albicans* susceptibility, do not translate to universal broad-spectrum antimicrobial efficacy. These observations align with earlier studies reporting limited activity of rosemary and other aromatic essential oils against *P. aeruginosa* and *Bacillus* species, which are frequently among the least susceptible bacteria in screening assays [50,51]. Collectively, these findings underscore the importance of strain- and state-specific susceptibility assessments in the antimicrobial evaluation of natural products.

**Figure 3:**
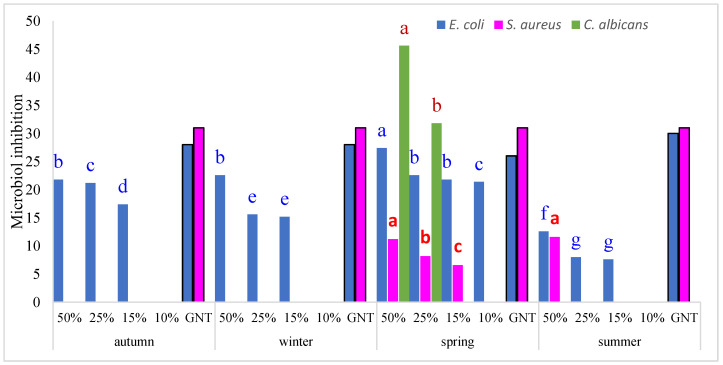
*Rosmarinus officinalis* essential oils, seasonal variation concentration-dependent variation in the antimicrobial activity against *Escherichia coli, Staphylococcus aureus*, *Candida albicans*. Bars represent mean ± SD (n = 3). Different letters indicate significant differ ences among means (*p* < 0.05, LSD test).

*Staphylococcus aureus* displayed weak sensitivity, inhibited only in summer (11 mm at 50%), far below Gentamicin’s consistent activity (31–33 mm). Remarkably, spring oils demonstrated strong antifungal activity against *Candida albicans*, with inhibition zones of 45 mm (50%) and 32 mm (25%). No antifungal effect was observed in other seasons, suggesting the spring-specific accumulation of antifungal phytochemicals. To validate these observations, a two-way ANOVA was conducted to assess the effects of season, concentration, and their interaction on microbial inhibition (Figure 3). The results showed that both season and concentration, as well as their interaction, significantly influenced the inhibition of *E. coli*, *S. aureus*, and *C. albicans* (*p* < 0.001). Post hoc LSD tests (*p* < 0.05) confirmed statistically distinct groupings among oils from different seasons, with spring samples showing the greatest inhibitory effect against *E. coli*, and spring oils displaying unique antifungal activity against *C. albicans*. By contrast, no activity was detected against *P. aeruginosa* and *B. subtilis* across all seasons and concentrations tested. Figure 3 illustrates these findings, showing clear seasonal and concentration-dependent differences: autumn and summer oils displayed the strongest antibacterial activity against *E. coli*, while spring oils were uniquely effective against *C. albicans*. Overall, these results highlight the critical role of seasonal harvest timing in determining the antimicrobial efficacy of *R. officinalis* EOs. Oils harvested in autumn and summer appear most suitable for antibacterial purposes, whereas spring harvests may be optimal for antifungal applications.

The concentrations used in this antimicrobial screening (50%, 25%, 15% and 10% *v*/*v*) were deliberately selected to enable preliminary comparative evaluation of seasonal effects using the disc diffusion assay. This method requires relatively high essential oil loads to produce measurable inhibition zones, as lipophilic compounds diffuse poorly through agar. Consequently, it serves as a qualitative screening tool rather than a quantitative determination of minimum inhibitory concentrations (MICs). Reported MIC values for *R. officinalis* essential oils vary widely depending on chemotype, strain, and assay type, from 0.20 to 0.48 mg mL^−1^ for Gram-positive bacteria and 1.16–1.72 mg mL^−1^ for Gram-negative strains, to as high as 11.25 mg mL^−1^ against *S. aureus* [52,53]. Assuming a representative oil density of 0.9 g mL^−1^, these MICs correspond approximately to 0.02–1.25% *v*/*v*—values 8–50 times lower than our lowest screening concentration. Such discrepancies arise from methodological differences between agar diffusion and broth microdilution, variation in oil composition and purity, and the absence of surfactants or solubilizers in our crude preparations, which restrict diffusion and may underestimate intrinsic activity [54,55,56].

Accordingly, the limited antibacterial activity observed at 10–15% (7–17 mm inhibition against *E. coli*) suggests that the oils possess only modest antibacterial potential under these test conditions, likely constrained by the low diffusibility of lipophilic constituents in agar media. Nevertheless, the consistently measurable inhibition zones in autumn, winter, and summer samples indicate that seasonal compositional changes do modulate antibacterial performance. In contrast, the pronounced antifungal effect of the spring oil against *C. albicans* (45 mm at 50%, 32 mm at 25%) represents a genuine and season-specific activity peak, supporting the hypothesis that certain oxygenated terpenes accumulate preferentially during this period. This zone diameter exceeds those typically reported for many commercial antifungal agents at standard concentrations, warranting dedicated investigation of spring oil fractionation to isolate the responsible constituents. These findings highlight that, while the overall antimicrobial efficacy of *R. officinalis* essential oils remains modest compared with conventional antibiotics, seasonal harvest timing still plays a critical role in shaping their bioactivity profiles.

From a practical standpoint, the concentrations tested here exceed those typically used in most applications. Food preservation systems generally employ essential oils at concentrations of ≤0.5% *v*/*v* to meet sensory and safety standards [57,58], whereas topical antiseptics or dermatological formulations may use concentrations of up to a few percent (commonly 0.1–5%), depending on the formulation and safety data [58]. Consequently, the modest antibacterial response observed in this study is unlikely to have direct relevance for food preservation but could inform further optimization for topical or cosmetic applications, particularly when combined with formulation strategies that enhance bioavailability. Future investigations should employ standardized broth microdilution assays (CLSI/EUCAST) to establish quantitative MIC and MBC values at lower concentrations (0.1–5% *v*/*v*), assess cytotoxicity on human keratinocyte or fibroblast lines to determine selectivity indices, and explore nanoemulsion or encapsulated formulations known to enhance dispersion and reduce effective dose requirements [59,60,61].

### 2.5. In Vito Anti-Inflammatory Activity

**Figure 4:**
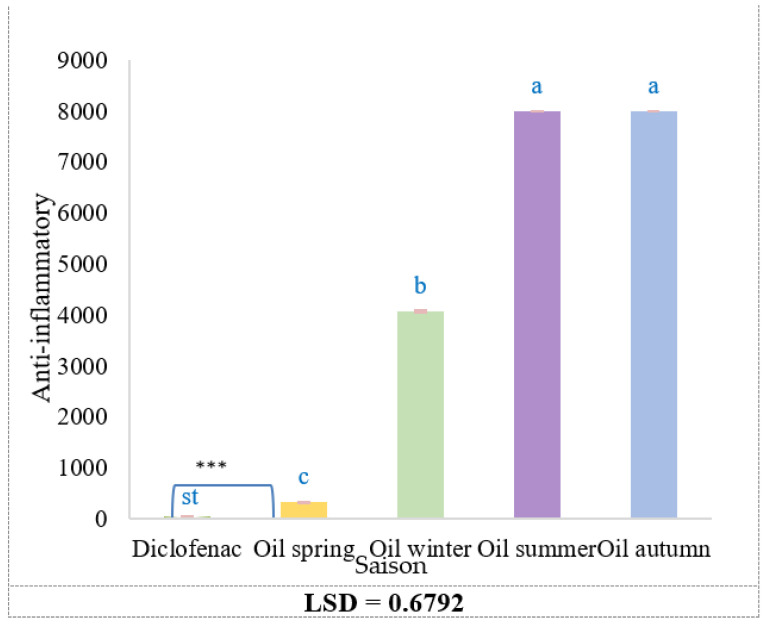
*R. officinalis* essential oils, seasonal variation, concentration-dependent variation in the anti-inflammatory activity. Bars represent mean ± SD (n = 3). Different letters denote statistically significant differences among means according to LSD test (*p* < 0.05). ***: Highly significant.

The anti-inflammatory activity of *Rosmarinus officinalis* essential oils (EOs) obtained in different seasons was assessed by measuring IC_50_ values, where lower values indicate higher activity (Table 5). Spring oil demonstrated the highest activity, with an IC_50_ of 326.54 ± 5.07 µg/mL, followed by winter oil (4076.22 ± 6.20 µg/mL). In contrast, summer and autumn oils exhibited negligible activity, with IC_50_ values exceeding 8000 µg/mL. The reference drug diclofenac, used as positive control, showed a markedly stronger activity (IC_50_ = 40.90 ± 0.89 µg/mL). Statistical analysis by One-way ANOVA revealed a highly significant seasonal effect on the anti-inflammatory activity of *R. officinalis* essential oils (*p* < 0.0001). The mean square for the seasonal factor (67,756,662.6) substantially exceeded the residual mean square (152.5), confirming the robustness of this variation. Post hoc LSD analysis (*p* < 0.05) further indicated that spring oils exhibited significantly greater inhibition of protein denaturation than samples from other seasons, consistent with their enhanced antioxidant potential. The least significant difference (LSD = 0.6792) enabled clear discrimination among seasonal treatments (Figure 4).

The superior activity of spring oil may be linked to its richer content in bioactive monoterpenes and phenolic compounds, which are known for their anti-inflammatory potential. In contrast, the weak activity of winter oil and the negligible activity of summer and autumn oils may reflect seasonal depletion of these key constituents. Although the spring oil demonstrated promising effects, its potency remained substantially lower than that of diclofenac. This suggests that further purification, enrichment, or formulation strategies may be required to enhance its therapeutic potential (see Table 5).

### 2.6. Acute Toxicity

The acute oral toxicity of *Rosmarinus officinalis* essential oil (EO) was assessed in rats at a single dose of 2000 mg/kg following OECD Guideline 423. No mortality or visible signs of systemic, neurological, or gastrointestinal toxicity were observed during the 14-day observation period. All animals maintained normal behavior, body-weight gain, and food and water consumption throughout the study.

These findings indicate that the EO has an estimated LD_50_ > 2000 mg/kg, placing it in Category 5 (low acute toxicity) according to the OECD and Globally Harmonized System (GHS) classifications. The absence of clinical or behavioral abnormalities confirms a wide margin of safety under the tested conditions.

The absence of overt toxicity is consistent with previous reports on rosemary essential oils and their major constituents, such as camphor, 1,8-cineole, and α-pinene, which are generally regarded as safe at moderate doses.

While these results demonstrate preliminary safety, further studies are warranted to confirm long-term tolerance, including sub-chronic, reproductive, and genotoxic evaluations, as well as cytotoxicity assessments on human cell lines to establish a comprehensive toxicological profile for pharmaceutical or nutraceutical use.

### 2.7. Statistical Correlation and Structure Activity Relationships

A correlation analysis was conducted to explore the relationships between the individual chemical constituents of *Rosmarinus officinalis* essential oils (EOs) and their observed biological activities across different harvest seasons. The results revealed distinct and informative structure–activity relationships (SARs), highlighting the significant role of both major and minor components in shaping the oils’ bioactivity profiles.

Interestingly, several minor constituents exhibited strong, often negative, correlations with antioxidant and anti-inflammatory activities, suggesting their disproportionate contribution to the overall bioactivity. For example, α-thujene, though present only in trace amounts (0.17–0.92%), showed strong negative correlations with DPPH (r = −0.995), ABTS (r = −0.988), and SNP (r = −0.987) assays. Likewise, α-phellandrene and α-calacorene correlated negatively with ADS and FRAP assays (r = −0.999 and r = −0.954, respectively), indicating their involvement in electron donation and radical stabilization processes. These findings support the concept that even low-abundance terpenes may play key roles in mitigating oxidative stress, consistent with previous reports emphasizing the synergistic or antagonistic effects among EO constituents [62,63].

Cuminic aldehyde also displayed robust negative correlations with all antioxidant assays (DPPH: r = −0.968; ABTS: r = −0.983; SNP: r = −0.983). Its conjugated aromatic and electrophilic structure facilitates resonance stabilization and nucleophilic interactions with reactive species, enhancing its radical-scavenging potential [64]. Similarly, γ-terpinene exhibited high correlations (DPPH: r = −0.961; ABTS: r = −0.975; SNP: r = −0.975), attributable to its hydrogen-donating and resonance-stabilizing properties as a 1,4-cyclohexadiene monoterpene [65].

Regarding anti-inflammatory activity (BSA denaturation assay), cuminic aldehyde again showed the strongest negative correlation (r = −0.995), followed by cis-β-terpineol (r = −0.984), γ-terpinene (r = −0.981), β-cis-ocimene (r = −0.978), and α-calacorene (r = −0.966). Compounds containing electrophilic carbonyl groups (e.g., cuminic aldehyde, α-citral) may inhibit protein denaturation through Michael-type addition to nucleophilic residues (cysteine, lysine, histidine), thereby stabilizing protein tertiary structures [66].

In contrast, the dominant constituents (1,8-cineole, α-pinene, camphor) showed weak or nonsignificant correlations with bioactivity parameters, emphasizing that overall efficacy results from the interactive network of components rather than a single major molecule [43,44,67]. The correlation with antibacterial activity was compound-specific, with β-myrcene (r = 0.988) and 1,8-cineole (r = 0.941) showing strong positive associations with *E. coli* inhibition, consistent with their membrane-disruptive properties.

**Figure 5:**
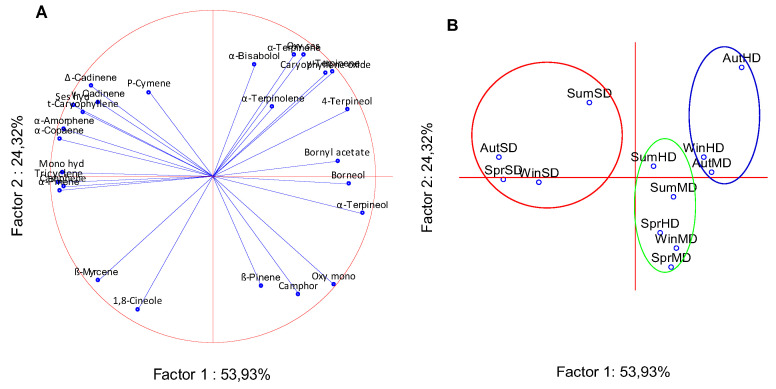
Chemical diversity of *R. officinalis* essential oils using the PCA approach. (**A**) plan (1/2) of variables; (**B**) plan (1/2) of cases.

In addition, to elucidate the relationship between extraction-dependent chemical diversity and biological properties, principal component analysis (PCA) was performed on the correlation matrix of 26 variables (compounds > 0.5%) across 12 essential oil samples. As the analysis was based on the correlation matrix, all variables were effectively standardized to have a mean of zero and a variance of one prior to decomposition. The PCA extracted 11 components, of which the first three (eigenvalues > 1) collectively accounted for 85.2% of the total variance (PC1 = 53.93%, PC2 = 24.32%, PC3 = 6.96%). According to the variance-threshold criterion proposed by Morrison [29], the retained components should explain at least 75% of the variance, a condition clearly met in the present analysis. The PC1–PC2 score plot (Figure 5) revealed three well-defined clusters reflecting both the extraction method and the seasonal variation.

Cluster I (negative PC1) comprised steam-distilled oils. It was characterized by elevated levels of mono- and sesquiterpene hydrocarbons, including tricyclene, α-pinene, camphene, β-myrcene, α-copaene, trans-caryophyllene, γ-cadinene, and δ-cadinene. Cluster II (positive PC1; AutHD, AutMD, WinHD) was enriched in oxygenated monoterpenes such as γ-terpinene, borneol, 4-terpineol, α-terpineol, bornyl acetate, and caryophyllene oxide. Cluster III (negative PC2; SumHD, SumMD, SprHD, SprMD, WinMD) was typified by high β-pinene, 1,8-cineole, and camphor contents and comparatively lower oxygenated sesquiterpenes. PC3 explained a limited amount of additional variance and did not contribute to further biologically distinct separation.

The PCA clustering patterns aligned strongly with bioactivity profiles. Spring oils from Cluster III exhibited the strongest antioxidant capacity (FRAP A_0.5_ = 4.63 µg/mL; DPPH IC_50_ = 39.22 µg/mL), while autumn and winter samples grouped in Cluster II displayed enhanced anti-inflammatory tendencies. However, correlation analysis indicated that these effects could not be attributed solely to the dominant terpenes. Several minor oxygenated constituents co-segregating within the clusters demonstrated exceptionally strong associations with antioxidant and anti-inflammatory endpoints (|r| > 0.823; *p* < 0.001), notably γ-terpinene (DPPH r = −0.961; ABTS r = −0.975; SNP r = −0.975; BSA r = −0.981), α-thujene (DPPH r = −0.995), cuminic aldehyde (BSA r = −0.995), α-phellandrene (FRAP r = −0.999), and α-calacorene (ADS r = −0.954). Conversely, hydrocarbon-rich Cluster I (steam-distilled oils) aligned with samples exhibiting greater antibacterial potential, consistent with the strong positive correlations between β-myrcene and *E. coli* inhibition (r = 0.988) and between 1,8-cineole and antibacterial activity (r = 0.941).

**Figure 6:**
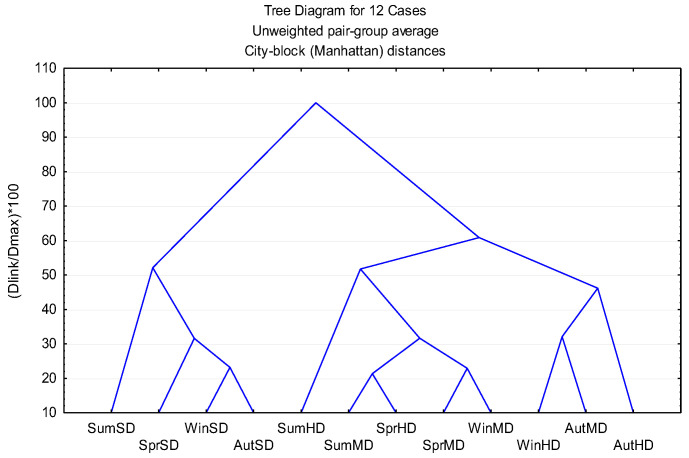
Dendrogram of *R. officinalis* essential oils, based on Manhattan Similarity distance.

Hierarchical clustering analysis (HCA) using Manhattan distance and UPGMA linkage (Figure 6) corroborated the PCA classification, revealing two principal clusters at linkage distances of approximately 4000–5000. Within the oxygenated-monoterpene cluster, spring extracts formed a distinct sub-cluster corresponding to the highest overall bioactivity, whereas autumn samples of the same extraction group exhibited comparatively weaker effects. The strong concordance between PCA and HCA confirms the statistical robustness of the observed grouping patterns.

Collectively, the correlation, PCA, and HCA analyses reveal that extraction technique is the primary determinant of chemical variance, while seasonal effects modulate the abundance of bioactivity-relevant oxygenated terpenes. The alignment between compositional clusters and functional responses provides robust evidence for composition–bioactivity coupling. Although supervised methods such as Partial Least Squares Regression (PLS-R) were not applied here, their integration in future studies could further quantify these predictive relationships.

These findings establish a coherent, statistically supported framework linking chemical diversity to biological performance. They underscore that the pharmacological potential of *R. officinalis* EOs arises from the concerted interplay of multiple constituents rather than from a single dominant component, offering a rational basis for optimizing harvest timing and extraction strategy.

A limitation of this study is that site-specific environmental parameters (soil properties, microclimate, water availability) were not systematically monitored during sampling. These factors are known to influence secondary metabolite profiles and may contribute to the observed seasonal variation [68]. Future work should integrate environmental profiling with phytochemical characterization to enable mechanistic modeling. In addition, while correlation, PCA and HCA provided valuable exploratory insights, the statistical treatment remains descriptive rather than predictive. More advanced supervised models (e.g., PLS-R or OPLS-DA) and hypothesis-driven significance testing will be incorporated in future work to quantitatively validate and strengthen these chemometric associations. Nevertheless, the robust concordance between multivariate clustering, correlation analysis, and bioactivity assays validates that seasonal timing reliably predicts functional properties for practical harvest optimization. These quantitative structure–activity relationships establish a predictive framework for rational valorization of *R. officinalis* essential oils in pharmaceutical and nutraceutical applications.

## 3. Materials and Methods

### 3.1. Plant Material and Sampling

The aerial parts of *Rosmarinus officinalis* L. (*Salvia rosmarinus* Spenn). were collected during the period 2022–2023, with sampling performed in the middle of each season (autumn, winter, spring and summer). The plant was taxonomically identified Pr. Djilani Ghemam Amara from the Department of biology, University of El Oued, Algeria, and a voucher specimen (code: Ros-offc-001-4-2022) was deposited in the Herbarium of the Laboratory of Biomass, ENS-Kouba, Algiers, Algeria. Plant material was sourced from the El Kef Lakhdar area in Medea province, located at an altitude of 1164 m (35°57′33″ N, 3°12′16″ E). Only healthy, mature plants growing under comparable environmental conditions were selected to minimize variability. Freshly harvested material was immediately transported in clean, airtight containers to prevent degradation and contamination. Samples were thoroughly washed with deionized water to remove dust and debris, then spread on clean trays and shade-dried at ambient temperatures (20–25 °C) for 15 days to prevent photodegradation of volatile constituents. The dried plant material was ground using an agate mortar and pestle or an electric grinder to achieve a uniform particle size (<200 µm). Powdered samples were stored in airtight containers under cool, dry conditions until further analysis. In total, twelve essential oil samples were obtained, with oils extracted by three different distillation methods for each season.

### 3.2. Chemicals and Reagents

All reagents and chemicals used in this study were of analytical grade to ensure high purity and reproducibility. Key reagents included Folin–Ciocalteu reagent (FCR), sodium carbonate (Na_2_CO_3_), DPPH (2,2-diphenyl-1-picrylhydrazyl), ABTS, α-tocopherol (vitamin E), BHT (2,6-di-tert-butyl-4-methylphenol), gallic acid, quercetin, aluminum chloride (AlCl_3_), phenanthroline, pancreatic α-amylase enzyme (1 U), acarbose, and soluble starch. These were purchased from Sigma-Aldrich (St. Louis, MO, USA). Standards such as α-tocopherol (C_29_H_50_O_2_), BHT (C_15_H_24_O), and quercetin (C_15_H_10_O_7_) were used as reference compounds for calibration and comparison. Anhydrous sodium sulfate (Na_2_SO_4_) was used for drying essential oils. Additional reagents, including iron (III) chloride (FeCl_3_), sodium bicarbonate (NaHCO_3_), potassium iodide (KI), potassium acetate (CH_3_CO_2_K), and potassium persulfate (K_2_S_2_O_8_), were obtained from Biochem Chemopharma (Cosne-Cours-sur-Loire, France). Antimicrobial assays were performed against laboratory reference strains obtained from the Institute Pasteur (Algiers, Algeria): *Staphylococcus aureus* ATCC 6538 and *Bacillus subtilis* ATCC 6633 (Gram-positive); *Pseudomonas aeruginosa* ATCC 9027 and *Escherichia coli* ATCC 8739 (Gram-negative); and *Candida albicans* ATCC 10231 (yeast). Filamentous fungi strains were provided by the National Museum of Natural History (Paris, France) via the Institute Pasteur, Algiers. All spectrophotometric and enzymatic activities were measured using a PerkinElmer Multimode Plate Reader EnSpire (Waltham, MA, USA) at the National Center for Biotechnology Research (Constantine, Algeria).

### 3.3. Essential Oil Extraction

The powdered aerial parts of *Rosmarinus officinalis* L. were subjected to three distinct extraction techniques, hydrodistillation (HD), steam distillation (SD), and microwave-assisted distillation (MD), to obtain the essential oils (EOs). For each seasonal batch, 100 g of dried plant material was placed in a 2 L round-bottom flask containing 1.5 L of distilled water (solid-to-liquid ratio 1:15 *w*/*v*) and connected to a Clevenger-type apparatus (VWR International, Radnor, PA, USA).

Hydrodistillation was performed by boiling the mixture under atmospheric pressure for 3 h, whereas steam distillation involved passing steam through the plant matrix for 3 h under identical conditions. For microwave-assisted distillation, the plant material was combined with 250 mL of distilled water (solid-to-liquid ratio 1:2.5 *w*/*v*) and subjected to microwave heating in a Midea AG823ABI oven (Foshan, China) operating at 800 W for 30 min.

At the end of each extraction, the collected distillates were decanted to separate the essential oil phase, which was subsequently dried over anhydrous sodium sulfate (Na_2_SO_4_, Sigma-Aldrich, St. Louis, MO, USA) to remove residual moisture. The purified oils were measured to determine yield (*v*/*w*, relative to the dry weight of plant material) and stored in sealed amber glass vials at 4 °C until subsequent analyses.

### 3.4. Essential Oil Analysis

The chemical composition of *Rosmarinus officinalis* essential oils (EOs) obtained using different extraction techniques was analyzed by gas chromatography with flame ionization detection (GC-FID) for quantification and gas chromatography–mass spectrometry (GC–MS) for compound identification.

GC-FID analysis was performed on a Shimadzu GC-2010 Plus system (Kyoto, Japan) equipped with an Rtx-5MS fused-silica capillary column (30 m × 0.25 mm i.d., 0.25 µm film thickness; Restek, Bellefonte, PA, USA). The oven temperature was initially set at 60 °C (5 min), increased at 3 °C min^−1^ to 250 °C, and held isothermally for 10 min. The injector and detector temperatures were maintained at 250 °C and 300 °C, respectively. The injection volume was 1 µL (split ratio 1:20), and nitrogen was used as the carrier gas at a constant flow rate of 1.0 mL min^−1^. Essential oil samples were diluted to 1% (*v*/*v*) in n-hexane prior to injection. Relative compound percentages were determined by electronic integration of GC-FID peak areas without correction factors.

GC–MS analysis was conducted on a Hewlett-Packard 6890 gas chromatograph coupled to a 5973A mass spectrometer (Agilent Technologies, Palo Alto, CA, USA) under identical chromatographic conditions, using a non-polar HP-5MS fused-silica capillary column (30 m × 0.25 mm i.d., 0.25 µm film thickness). The oven program was: 60 °C (8 min), ramped at 2 °C min^−1^ to 250 °C, and held for 15 min. Helium served as the carrier gas at a constant flow rate of 1.5 mL min^−1^. The injector temperature was 250 °C (split ratio 1:20), with an injection volume of 1 µL. The MS was operated in electron impact (EI) mode at 70 eV, with an ion source temperature of 200 °C, electron multiplier voltage of 1800 V, and mass scan range of *m/z* 40–400.

Compound identification was achieved by comparing obtained mass spectra with those in the NIST (National Institute of Standards and Technology) and Wiley libraries. Identification was further confirmed by calculating Kovàts retention indices (RI) relative to a homologous series of n-alkanes (C_5_–C_28_) analyzed under identical conditions and comparing these with literature data [69]. Quantification of individual constituents was based on GC-FID peak-area normalization and expressed as relative percentages of the total essential oil composition.

### 3.5. Antioxidant Activity In Vitro

The antioxidant potential of *Rosmarinus officinalis* essential oils (EOs) obtained by different extraction methods and seasons was assessed using five complementary assays: DPPH and ABTS radical scavenging, ferric reducing antioxidant power (FRAP), superoxide radical scavenging (alkaline DMSO method), and silver nanoparticle (AgNP) reduction. All assays were performed in triplicate, and absorbance was measured against reagent blanks containing all components except the test sample. Results were expressed as IC_50_ (µg/mL) or A_0.5_ (µg/mL) values [70].

#### 3.5.1. DPPH Radical Scavenging

The DPPH radical–scavenging activity of the essential oils was evaluated following the method of Brand-Williams et al. [13], with minor modifications. In a 96-well microplate, 160 µL of a freshly prepared 0.1 mM DPPH solution in methanol was mixed with 40 µL of EO sample or standard antioxidant (BHA). The mixtures were incubated for 30 min in the dark at ambient temperature, and absorbance was recorded at 517 nm using a microplate reader. Radical inhibition (%) was calculated as:

I% _(DPPH)_= ((A_0_ − A_1_)/A_0_) × 100 (1)

where A_0_ = control absorbance and A_1_ = sample absorbance, and IC_50_ values were determined from the inhibition curves.

#### 3.5.2. ABTS Radical Scavenging

The ABTS assay was conducted according to Re et al. [71], adapted to microplate format. The ABTS•^+^ radical was generated by mixing 2 mM ABTS with 2.45 mM potassium persulfate and allowing the mixture to stand for 16 h at room temperature in the dark. The resulting solution was diluted with methanol to an absorbance of 0.700 ± 0.025 at 734 nm. For the assay, 40 µL of EO sample or standard (BHA) was mixed with 160 µL of ABTS•^+^ solution in each well, incubated for 10 min at 25 °C, and absorbance was read at 734 nm. IC_50_ values were obtained from inhibition curves.

#### 3.5.3. Ferric Reducing Antioxidant Power (FRAP) Assay

The ferric reducing capacity of the EOs was determined using the potassium ferricyanide method of [72], adapted to microplate conditions. Ten µL of EO solution (3.125–200 µg/mL) was combined with 40 µL of 0.2 M phosphate buffer (pH 6.6) and 50 µL of 1% potassium ferricyanide. The mixture was incubated at 50 °C for 20 min, followed by the addition of 50 µL of 10% trichloroacetic acid, 40 µL of distilled water, and 10 µL of 0.1% ferric chloride. Absorbance was measured at 700 nm. Reducing power was expressed as A_0.5_, the concentration yielding an absorbance of 0.5, and compared to ascorbic acid standards.

#### 3.5.4. Superoxide Radical Scavenging (Alkaline DMSO Method)

Superoxide radical–scavenging activity (ADS assay) was measured using the alkaline DMSO method [73], adapted for essential oils. In each well, 30 µL of nitroblue tetrazolium (NBT, 1 mg/mL), 40 µL of EO sample or tannic acid standard, and 130 µL of freshly prepared alkaline DMSO (0.1 M NaOH, pH 12.5) were combined. The reaction mixtures were incubated for 5 min at 25 °C, and absorbance was read at 560 nm. A lower absorbance indicated stronger scavenging activity.

#### 3.5.5. Silver Nanoparticle (AgNP) Reduction Assay

The antioxidant capacity of the EOs was further assessed using the silver nanoparticle reduction method of Özyürek et al. (2012) [74]. Fresh AgNP reagent was prepared by reducing 1 mM AgNO_3_ with trisodium citrate. For the assay, 130 µL of AgNP reagent, 50 µL of distilled water, and 20 µL of EO sample or ascorbic acid standard were mixed in a 96-well plate. The mixtures were incubated for 30 min at 25 °C in the dark, and absorbance was measured at 423 nm. Antioxidant capacity was expressed as the concentration producing A_0.5_, relative to standard antioxidants.

### 3.6. Anti-Inflammatory Activity In Vitro

The anti-inflammatory potential of *R. officinalis* essential oils was evaluated by the bovine serum albumin (BSA) denaturation inhibition assay, as described by Karthik and Thangaswamy [75]. Briefly, 1 mL of EO sample was mixed with 1 mL of 0.2% BSA in 0.05 mM Tris-HCl buffer (pH 6.6). The mixture was incubated at 37 °C for 15 min, heated to 72 °C for 5 min, and then cooled to room temperature. Absorbance was measured at 660 nm using a UV–Vis spectrophotometer (Shimadzu, Kyoto, Japan). Diclofenac sodium and ketoprofen served as positive controls. All experiments were performed in triplicate. The percentage inhibition of protein denaturation was calculated from absorbance changes and expressed as inhibition percentage (I%).

### 3.7. Antibacterial and Antifungal Activities In Vitro

The antimicrobial activity of *Rosmarinus officinalis* essential oils was evaluated using the agar disc diffusion method following established protocols for essential oil testing [54,76]. Five reference strains were tested: *Escherichia coli* ATCC 8739, *Pseudomonas aeruginosa* ATCC 9027, *Staphylococcus aureus* ATCC 6538, *Bacillus subtilis* ATCC 6633, and *Candida albicans* ATCC 10231.

Briefly, overnight microbial cultures grown at 37 °C were adjusted to 0.5 McFarland standard (approximately 10^8^ CFU/mL) in sterile saline. One hundred microliters of each suspension were spread evenly onto Mueller–Hinton agar (for bacteria) or Sabouraud dextrose agar (for yeast) plates. Sterile 6 mm Whatman No. 1 filter discs were impregnated with 10 µL of essential oil solution and placed on the inoculated plates. Essential oils were tested at concentrations of 50%, 25%, 15% and 10% (*v*/*v*) diluted in dimethyl sulfoxide (DMSO). Negative controls (DMSO alone) and positive controls, gentamicin (10 µg/disc) for bacteria and nystatin for *C. albicans*, were included. Plates were incubated at 37 °C for 18–24 h for bacteria and 48 h for yeast, after which inhibition zone diameters (mm) were measured with calipers, including the disc diameter.

These relatively high concentrations were selected to enable comparative screening of seasonal essential oils using the disc diffusion method, which requires higher essential oil loads than broth microdilution because lipophilic compounds diffuse poorly through agar media [54]. Comparable concentration ranges (25–100%) have been employed in previous essential oil antimicrobial studies using the same assay format [77,78,79,80].

### 3.8. Acute Toxicity

Acute oral toxicity was evaluated in rats according to OECD guideline 423. Four groups of five animals each were used. Rats in the treatment groups received a single oral administration of 0.5 mL of the EO, corresponding to 2000 mg/kg body weight, while the control group received an equivalent volume of 0.9% physiological saline.

Following administration, animals were observed continuously for the first 4 h to detect any immediate adverse effects before resuming access to food and water. Behavioral and clinical signs were carefully monitored, including hyperactivity, ataxia, tremors, convulsions, seizures, salivation, diarrhea, lethargy, somnolence, and coma. Mortality was also recorded. Daily monitoring continued for 14 days to detect delayed toxicity, with attention to body-weight changes, food and water intake, and general clinical condition.

At the end of the observation period, the absence or presence of mortality and abnormal clinical signs was recorded for subsequent evaluation of the median lethal dose (LD_50_) according to OECD classification criteria.

### 3.9. Statistical Analysis

All experiments were performed in triplicate, and results are expressed as mean ± standard deviation (SD). Statistical analyses were conducted using SAS software, version 9.1 (SAS Institute Inc., Cary, NC, USA, 2008). One-way and two-way analysis of variance (ANOVA) was applied to assess the effects of season and extraction method on essential oil composition and biological activities. Post hoc comparisons were performed using Tukey’s test at a significance level of *p* < 0.05. The ANOVA models included both main effects and two-way interactions of the studied factors. Statistically homogeneous groups were identified using least significant difference (LSD) values, with identical letters denoting no significant difference among means. In addition, descriptive statistics were generated using STATISTICA software (TIBCO Software Inc., Palo Alto, CA, USA) to summarize the dataset and provide preliminary insights. Results tables report the key outputs of the variance analyses, including *p*-values from F-Snedecor (Fisher–Snedecor) tests, which quantify the influence of the experimental factors.

## 4. Conclusions

This study demonstrates that both harvest season and extraction technique significantly affect the yield, chemical composition, and biological activities of *Rosmarinus officinalis* essential oils. The spring harvest produced the highest yield (0.81% via hydrodistillation) and the most diverse chemical profile, dominated by camphor (up to 36.52%) and α-pinene (up to 21.72%). Among the tested techniques, microwave-assisted distillation (MD) proved most effective for preserving oxygenated monoterpenes, compounds closely associated with antioxidant and antimicrobial efficacy. Spring oils, particularly those obtained by MD, exhibited superior antioxidant capacity (IC_50_ = 39.22 µg/mL for DPPH; 30.71 µg/mL for ABTS). They also showed strong antimicrobial effects against *Staphylococcus aureus*, *Bacillus subtilis*, and *Candida albicans*, while acute toxicity testing indicated safety up to 2000 mg/kg, supporting their potential for nutraceutical and pharmaceutical use. Future work should explore genomic and metabolic profiling to elucidate the seasonal regulation of bioactive compound synthesis and the scale-up of solvent-free microwave-assisted extraction (SFME) as a sustainable industrial method. Moreover, incorporating *R. officinalis* essential oils into advanced drug delivery systems, green nanotechnology platforms, and functional food formulations could expand their therapeutic and commercial applications.

## Figures and Tables

**Table 1:** The chemical composition of essential oil *Rosmarinus officinalis* L. wild-grown (aerial parts), where KI represents the retention index.

Seasons	Empirical Formula	TR	IR	Autumn			Winter			Spring			Summer		
Extraction Method				HD (%)	MD (%)	SD (%)	HD (%)	MD (%)	SD (%)	HD (%)	MD (%)	SD (%)	HD (%)	MD (%)	SD (%)
Constituent															
Tricyclene	C_10_H_16_	4.37	922	0.54	0.74	1.47	0.73	0.87	1.33	0.92	0.64	1.62	0.83	0.67	0.9
α-Thujene	C_10_H_16_	4.52	924	0.83	0.92		0.83	0.3		0.31	0.17		0.25	0.23	0.3
α-Pinene	C_10_H_16_	4.74	926	7.31	9.2	18.27	10.31	12.08	18.62	12.31	11.29	21.72	15.88	12.89	14.64
Camphene	C_10_H_16_	5.19	942	7.84	9.36	16.3	9.88	10.71	15.11	12.35	10.42	18.23	14.86	12.58	13.28
Sabinene	C_10_H_16_	6.03	971	1.79	/	/	/	/	/	/	0.18	/	/	/	/
β-Pinene	C_10_H_16_	6.13	974	1.92	2.66	1.73	3.35	3.05	2.26	5.02	4.32	2.33	1.61	1.68	1.67
β-Myrcene	C_10_H_16_	6.79	988	0.37	0.48	0.74	0.59	0.73	0.72	0.66	0.66	0.75	0.53	0.63	0.57
α-Phellandrene	C_10_H_16_	7.33	1001	0.28	0.21	0.5	0.3	0.23	0.24	0.3	0.27	0.37	0.4	0.46	0.45
α-Terpinene	C_10_H_16_	7.98	1020	1.34	1.14	0.75	1.31	0.66	0.66	0.66	0.66	0.87	1.05	1.12	1.09
*p*-Cymene	C_10_H_16_	8.48	1023	3.02	3.25	3.98	2.98	2.35	4	2.35	0.29	3.07	2.8	3.07	2.49
1,8-Cineole	C_10_H_18_O	8.86	1027	7.65	8.5	12.54	10.96	12.84	13.49	13.96	14.53	13.59	10.06	11.73	9
cis-Ocimene	C_10_H_16_	9.34	1034	0.18	0.2	0.18	0.15	0.12	/	0.26	0.26	/	0.12	0.14	0.14
trans-β-Ocimene	C_10_H_16_	9.97	1045	/	/	/	/	/	/	0.1	0.13	/	0.04	0.04	/
γ-Terpinene	C_10_H_16_	10.46	1103	2.33	1.68	0.73	1.88	0.86	0.57	0.99	0.82	0.75	1.21	1.32	1.27
cis-Sabinene hydrate	C_10_H_18_O	11.13	1112	0.63		0.19	0.43	0.41	0.15	0.23	0.36		0.13	0.21	0.17
α-Terpinolene	C_10_H_16_	12.21	1120	1.05	0.72	0.37	0.78	0.54	0.32	0.53	0.5	0.43	0.71	0.79	0.72
cis-β-Terpineol	C_10_H_18_O	13.11	1138	0.58	1.02		0.32	0.3		0.18	0.26	/	0.11	0.15	0.12
Linalool	C_10_H_18_O	13.78	1141	/	/	/	2	0.2	/	0.15	0.24	/	0.08	0.09	0.1
Camphor	C_10_H_16_O	16.16	1158	23.53	30.78	22.92	24.48	33.63	22.91	33.03	36.52	21.95	31.29	35.37	24.13
Borneol	C_10_H_18_O	17.7	1176	5.3	6.57	1.42	5.44	6.52	3.56	3.77	3.46	1.49	3.66	4.07	3.11
4-Terpineol	C_10_H_18_O	18.42	1191	6.48	4.63	0.82	3.93	2.61	0.97	2.11	2.04	0.59	1.73	2.11	1.47
α-Terpineol	C_10_H_18_O	19.34	1203	2.34	2.38	0.79	2.61	2.72	0.99	2.22	2.13	0.58	1.52	1.82	1.17
Cuminic aldehyde	C_10_H_12_O	22.24	1238	0.13	0.13		0.08	/	/	/	/	/	/	/	/
Bornyl acetate	C_12_H_20_O_2_	25.38	1285	1.38	1.41	0.68	1.29	1.4	0.96	1.17	1.17	0.71	1.46	1.71	1.47
Thymol	C_10_H_14_O	27.21	1290	/	0.08	/	0.09	0.07	/	0.05	0.05	/	/	/	/
Carvacrol	C_10_H_14_O	28.08	1299		0.18		0.13	0.07		0.06	0.06		0.13	0.07	0.06
α-Terpinyl acetate	C_12_H_20_O_2_	29.62	1347	1.03	0.57		1.65	0.11		0.05	0.05		0.1	0.1	0.07
Eugenol	C_10_H_12_O_2_	30.39	1358				0.31								
α-Ylangene	C_15_H_24_	30.56	1373	0.12	0.12	0.4	0.16	0.12	0.29	0.11	0.11	0.35	0.11	0.12	0.26
α-Copaene	C_15_H_24_	30.84	1375	0.31	0.38	1.5	0.38	0.32	1.25	0.26	0.34	1.26	0.32	0.33	0.92
β-Elemene	C_15_H_24_	32.05	1391				0.09								
Eugenol methyl ether	C_11_H_14_O_2_	33.46	1410				1.77								
trans-Caryophyllene	C_15_H_24_	33.42	1418	0.98	0.08	2.5	0.1	0.5	1.47	0.76	0.76	1.99	1.03	0.95	2.85
α-Humulene	C_15_H_24_	32.62	1449			0.33		0.06	0.24	0.08	0.08	0.23	0.11	0.09	0.36
trans-β-Farnesene	C_15_H_24_	36.45	1453	0.07	0.06	0.22	0.06	0.06	0.2	0.06	0.05	0.15	0.08	0.07	0.2
α-Amorphene	C_15_H_24_	37.12	1484	0.33	0.3	1.75	0.33	0.24	1.16	0.34	0.32	1.1	0.3	0.28	1.12
α-Curcumene	C_15_H_22_	35.02	1489			0.17	0.04	0.02	0.13	0.03	0.03	0.09			
β-Selinene	C_15_H_24_	34.63	1491			0.13			0.19			0.17			
α-Muurolene	C_15_H_24_	38.59	1493	0.13	0.13	0.24	0.16	0.10	0.17	0.1	0.1	0.15	0.06	0.04	0.58
γ-Cadinene	C_15_H_24_	39.29	1513	0.32	0.25	0.72	0.26	0.20	0.56	0.22	0.2	0.96	0.13	0.11	1.31
δ-Cadinene	C_15_H_24_	39.9	1517	1.8	1.22	3.55	0.87	0.60	2.87	0.61	0.52	2.13	0.7	0.65	2.9
trans-Cadina-1,4-diene	C_15_H_24_	37.58	1533			0.23	0.06	0.03	0.17	0.05	0.04	0.15	0.04	0.03	0.22
α-Calacorene	C_15_H_20_	40.93	1546	0.34	0.14	0.4	0.15	0.04	0.35	0.06	0.03	0.22	0.09	0.06	0.35
Elemicin	C_12_H_16_O_3_	42.62	1555				0.10								
Caryophyllene oxide	C_15_H_24_O	43.09	1560	2.26	1.53	0.26	1.00	0.30	0.14	0.39	0.23		0.57	0.51	0.51
α-Cadinol	C_15_H_26_O	47.34	1651	0.32	0.06		0.40	0.07		0.25	0.13		0.21	0.08	0.2
α-Bisabolol	C_15_H_26_O	49.24	1684	2.86	0.52	0.84	1.70	0.60	1.16	1.96	0.76	0.92	2.14	0.98	2.37
Monoterpene hydrocarbons				28.8	30.56	45.02	33.09	32.5	43.83	36.76	30.61	50.14	40.29	35.62	37.52
Oxygenated monoterpene				46.64	54.27	38.68	50.78	59.37	42.07	55.76	59.65	38.2	48.71	55.62	39.33
Sesquiterpene hydrocarbons				4.4	2.68	12.14	2.66	2.29	9.05	2.68	2.58	8.95	2.97	2.73	11.07
Oxygenated sesquiterpene				5.44	2.11	1.1	3.1	0.97	1.3	2.6	1.12	0.92	2.92	1.57	3.08
Others				2.41	1.98	0.68	4.81	1.51	0.96	1.22	1.22	0.71	1.56	1.81	1.54
**Total amount of compounds**				87.69	91.6	97.62	94.44	96.64	97.21	99.02	95.18	98.92	96.45	97.35	92.54
Extraction yield (%)				0.76	0.50	0.66	0.45	0.25	0.32	0.81	0.65	0.78	0.63	0.43	0.55

**Table 2:** Comparison of our data with literature, (HD: Hydro distillation, MD: Microwave distillation, SD: Steam distillation).

Constituent	Country	Our Data (Algeria)	Italy	Brazil	South Africa	Morocco	Pakistan	Tunisia	Mexico	India. Kashmir	Iraq	Jordan	Turkey
	references	This study	[23]	[3]	[25]	[13]	[7]	[26]	[22]	[27]	[28]	[29]	[30]
	Extraction Method Used	HD/MD/SD	HD	HD	HD/MD	HD/MD	HD	HD	HD	HD	SD	SD	HD
	Seasons/Month Harvest	Autumn/Winter/Spring/Summer	Autumn/Winter/Spring/Summer	September	January	May	November–December (Winter)	Spring	ND	July	August	May	ND
	Ratio	(%)	(%)	(%)	(%)	(%)	(%)	(%)	(%)	(%)	(%)	(%)	(%)
**α-Pinene**		7.31–21.72	25.6–30.2	9.5	5.7/8.14	15.82/15.4	12.3	8.83	9.43	16.33	7.41	1.7	ND
**Camphene**		7.84–18.23	7.4–7.7	4	11.47/3.13	9.77/9.16	6	2.36	4.97	9.28	2.51	ND	22.45
**β-Pinene**		1.61–5.02	0.8–3.1	7	1.12/1.06	3.56/3.72	0.2	2.82	4.75	5.97	1.17	ND	ND
**1.8-Cineole**		7.65–14.53	17.2–19.1	14	11.91/10.56	31.2/32.18	38.5	57.55	1.56	14.33	21.24	14.6	35.36
**Camphor**		21.95–36.52	4.9–11.3	33.20	16.89/16.57	16.54/16.2	17.1	8.82	39.46	22.0	10.81	21.7	10.80
**Borneol**		1.42–6.57	7.1–8.4	ND	5.74/5.85	1.47/1.64	3.25	5.54	2.79	3.35	3.87	ND	8.26
**4-Terpineol**		0.59–6.48	1.5–1.7	1.0	1.42/1.56	7.16/7.36	3.25	1.02	1.18	1.115	5.14	5.2	ND

ND: not detected.

**Table 3:** Antioxidant activity of *R. officinalis* essential oils (IC_50_ and A_0.5_, µg/mL).

Products	IC_50_ (µg/mL)			A_0.5_ (µg/mL)	
	DPPH (μg/mL)	ABTS (μg/mL)	ADS (μg/mL)	FRAP (μg/mL)	SNP (μg/mL)
**Oil spring**	39.22 ± 3.01 ^c^	30.71 ± 0.38 ^c^	45.58 ± 0.44 ^b^	4.63 ± 0.29 ^b^	19.06 ± 1.60 ^c^
**Oil winter**	148.96 ± 3.81 ^b^	240.54 ± 2.91 ^b^	1215 ± 3.12 ^a^	300 ± 1.12 ^a^	123.09 ± 0.57 ^b^
**Oil summer**	1280 ± 5.82 ^a^	1310 ± 4.12 ^a^	1311 ± 4.127 ^a^	324 ± 1.14 ^a^	617 ± 1.14 ^a^
**Oil autumn**	1210 ± 5.12 ^a^	1207 ± 5.12 ^a^	1224 ± 5.15 ^a^	313 ± 1.15 ^a^	603 ± 1.14 ^a^
BHA *	6.89 ± 0.12	1.91 ± 0.09	NT	NT	NT
Tannic acid *	NT	NT	3.125 ± 0105	NT	NT
Ascorbic acid *	NT	NT	NT	6.77 ± 1.15	7.14 ± 0105

*: Standard compounds. Values are expressed as means ± S.D. of three parallel measurements. Means with different superscript letters in the same column (a,b,c) are significant (*p* < 0.05). NT: not tested.

**Table 4:** Antimicrobial activity of *R. officinalis* essential oils (zone of inhibition, mm).

	Strains Used	Microbial Inhibition					Seasons
		50%	25%	15%	10%	GNT	
**Gram-positive**	***Escherichia coli*** **ATCC 25922**	21	21	17	NI	27	Autumn
		22	15	15	NI	27	Winter
		7	9	7	NI	26	Spring
		27	21	13	NI	26	Summer
	***Pseudomonas aeruginosa*** **ATCC 27853**	NI	NI	NI	NI	26	Autumn
		NI	NI	NI	NI	26	Winter
		NI	NI	NI	NI	26	Spring
		NI	NI	NI	NI	26	Summer
**Gram-negative**	***Staphylococcus aureus*** **ATCC 25932**	NI	NI	NI	NI	31	Autumn
		NI	NI	NI	NI	32	Winter
		NI	NI	NI	NI	33	Spring
		11	NI	NI	NI	31	Summer
	***Bacillus subtilis*** **ATCC 25973**	NI	NI	NI	NI	24	Autumn
		NI	NI	NI	NI	24	Winter
		NI	NI	NI	NI	24	Spring
		NI	NI	NI	NI	23	Summer
**Yeast**	***Candida albicans*** **ATCC 10231**	NI	NI	NI	NI	/	Autumn
		NI	NI	NI	NI	/	Winter
		45	32	NI	NI	/	Spring
		NI	NI	NI	NI	/	Summer

NI = No Inhibition, GNT = Gentamicin.

**Table 5:** Anti-inflammatory activity of *R. officinalis* essential oils.

Oils Seasons	IC_50_ (µg/mL)
**Oil spring**	326.54 ± 5.07 ^c^
**Oil winter**	4076.223 ± 6.2 ^b^
**Oil summer**	8112 ± 5.2 ^a^
**Oil autumn**	8043 ± 5.2 ^a^
**Diclofenac**	40.90 ± 0.89

Values are expressed as means ± S.D. of three parallel measurements. Means with different superscript letters in the same column are significant (*p* < 0.05).

## Data Availability

The original contributions presented in the study are included in the article, further inquiries can be directed to the corresponding author.

## References

[1] Yeddes W., Aidi Wannes W., Hammami M., Smida M., Chebbi A., Marzouk B., Saidani Tounsi M. (2018). Effect of Environmental Conditions on the Chemical Composition and Antioxidant Activity of Essential Oils from *Rosmarinus officinalis* L. Growing Wild in Tunisia. J. Essent. Oil Bear. Plants.

[2] Aggoun K., Harkati B., Demirtaş I., Adem Ş., Jafari D.A., Hameed Z.A., Laouer H., Atoki A.V., Gouasmia A., Atanassova M. (2025). *Turgenia latifolia* (L.) Hoffm: Chemical Profiling and Antioxidant, Anti-Inflammatory, and Anticancer Activities. Bull. Chem. Soc. Ethiop..

[3] Fung Boix Y., Pimentel Victório C., Luiz Salgueiro Lage C., Machado Kuster R. (2010). Volatile Compounds from *Rosmarinus officinalis* L. and *Baccharis dracunculifolia* DC. Growing in Southeast Coast of Brazil. Quím. Nova.

[4] Yesil-Celiktas O., Sevimli C., Bedir E., Vardar-Sukan F. (2010). Inhibitory Effects of Rosemary Extracts, Carnosic Acid and Rosmarinic Acid on the Growth of Various Human Cancer Cell Lines. Plant Foods Hum. Nutr..

[5] Kant R., Kumar A. (2022). Review on Essential Oil Extraction from Aromatic and Medicinal Plants: Techniques, Performance and Economic Analysis. Sustain. Chem. Pharm..

[6] Boutekedjiret C., Bentahar F., Belabbes R., Bessiere J.M. (2003). Extraction of Rosemary Essential Oil by Steam Distillation and Hydrodistillation. Flavour Fragr. J..

[7] Hussain A.I., Anwar F., Chatha S.A.S., Jabbar A., Mahboob S., Nigam P.S. (2010). *Rosmarinus officinalis* Essential Oil: Antiproliferative, Antioxidant and Antibacterial Activities. Braz. J. Microbiol..

[8] Lucchesi M.E., Chemat F., Smadja J. (2004). An Original Solvent Free Microwave Extraction of Essential Oils from Spices. Flavour Fragr. J..

[9] Bozin B., Mimica-Dukic N., Samojlik I., Jovin E. (2007). Antimicrobial and Antioxidant Properties of Rosemary and Sage (*Rosmarinus officinalis* L. and *Salvia officinalis* L., Lamiaceae) Essential Oils. J. Agric. Food Chem..

[10] Bousbia N., Vian M.A., Ferhat M.A., Petitcolas E., Meklati B.Y., Chemat F. (2009). Comparison of Two Isolation Methods for Essential Oil from Rosemary Leaves: Hydrodistillation and Microwave Hydrodiffusion and Gravity. Food Chem..

[11] Mateus E.M., Lopes C., Nogueira T., Lourenço J.A.A., Curto M.J.M. (2006). Pilot Steam Distillation of Rosemary (*Rosmarinus officinalis* L.) from Portugal. Silva Lusit..

[12] Arafa G.K. (2019). Extract the Aromatic Oil of the Rosemary Plant by Steam Distillation and Hydro-Distillation Methods. Misr J. Agric. Eng..

[13] Elyemni M., Louaste B., Nechad I., Elkamli T., Bouia A., Taleb M., Chaouch M., Eloutassi N. (2019). Extraction of Essential Oils of *Rosmarinus officinalis* L. by Two Different Methods: Hydrodistillation and Microwave Assisted Hydrodistillation. Sci. World J..

[14] Sharifi-Rad M., Panda J., Mohanta Y.K., Pohl P., Zengin G., Moloney M.G. (2025). Essential oil of Cleome coluteoides (Boiss.): Phytochemical constituents, antioxidant, antimicrobial, antiproliferative, anti-inflammatory, enzymatic inhibition, and Xanthine oxidase inhibitory properties. J. Herb. Med.

[15] Sharifi-Rad M., Pohl P., Epifano F., Zengin G., Jaradat N., Messaoudi M. (2022). *Teucrium polium* (L.): Phytochemical Screening and Biological Activities at Different Phenological Stages. Molecules.

[16] Serralutzu F., Stangoni A., Amadou B., Tijan D., Re G.A., Marceddu S., Dore A., Bullitta S. (2020). Essential Oil Composition and Yield of a *Rosmarinus officinalis* L. Natural Population with an Extended Flowering Season in a Coastal Mediterranean Environment and Perspectives for Exploitations. Genet. Resour. Crop Evol..

[17] Lakušić D., Ristić M., Slavkovska V., Lakušić B. (2013). Seasonal Variations in the Composition of the Essential Oils of Rosemary (*Rosmarinus officinalis*, Lamiaceae). Nat. Prod. Commun..

[18] Daussy J., Staudt M. (2020). Do Future Climate Conditions Change Volatile Organic Compound Emissions from Artemisia Annua? Elevated CO_2_ and Temperature Modulate Actual VOC Emission Rate but Not Its Emission Capacity. Atmos. Environ. X.

[19] Ben Arfa A., Gouja H., Hannachi H., Isoda H., Neffati M., Najjaa H. (2022). Seasonal Changes in Rosemary Species: A Chemotaxonomic Assessment of Two Varieties Based on Essential Oil Compounds, Antioxidant and Antibacterial Activities. PLoS ONE.

[20] Bejenaru L.E., Biţă A., Mogoşanu G.D., Segneanu A.-E., Radu A., Ciocîlteu M.V., Bejenaru C. (2024). Polyphenols Investigation and Antioxidant and Anticholinesterase Activities of *Rosmarinus officinalis* L. Species from Southwest. Romania Flora. Molecules.

[21] Aziz Z.A.A., Ahmad A., Setapar S.H.M., Karakucuk A., Azim M.M., Lokhat D., Rafatullah M., Ganash M., Kamal M.A., Ashraf G.M. (2018). Essential Oils: Extraction Techniques, Pharmaceutical and Therapeutic Potential—A Review. Curr. Drug Metab..

[22] Silva-Flores P.G., Pérez-López L.A., Rivas-Galindo V.M., Paniagua-Vega D., Galindo-Rodríguez S.A., Álvarez-Román R. (2019). Simultaneous GC-FID Quantification of Main Components of *Rosmarinus officinalis* L. and Lavandula Dentata Essential Oils in Polymeric Nanocapsules for Antioxidant Application. J. Anal. Methods Chem..

[23] Melito S., Petretto G.L., Chahine S., Pintore G., Chessa M. (2019). Seasonal Variation of Essential Oil in Rosmarinus Officinalis Leaves in Sardinia. Nat. Prod. Commun..

[24] Sun W., Shahrajabian M.H. (2023). Therapeutic Potential of Phenolic Compounds in Medicinal Plants—Natural Health Products for Human Health. Molecules.

[25] Okoh O.O., Sadimenko A.P., Afolayan A.J. (2010). Comparative Evaluation of the Antibacterial Activities of the Essential Oils of *Rosmarinus officinalis* L. Obtained by Hydrodistillation and Solvent Free Microwave Extraction Methods. Food Chem..

[26] Yeddes W., Chalghoum A., Aidi-Wannes W., Ksouri R., Saidani Tounsi M. (2019). Effect of Bioclimatic Area and Season on Phenolics and Antioxidant Activities of Rosemary (*Rosmarinus officinalis* L.) Leaves. J. Essent. Oil Res..

[27] Tantry M.A., Shabir S., Khan R., Habib A., Akbar S. (2012). Determination of Essential Oil Composition of *Rosmarinus officinalis* Growing as Exotic Species in Kashmir Valley. Chem. Nat. Compd..

[28] Al Jaafreh A.M. (2024). Evaluation of Antioxidant Activities of Rosemary (*Rosmarinus officinalis* L.) Essential Oil and Different Types of Solvent Extractions. Biomed. Pharmacol. J..

[29] Morrison J.F., Tsai H.M., Bradshaw P. (1988). Conditional-sampling schemes for turbulent flow, based on the variable-interval time averaging (VITA) algorithm. Exp. Fluids.

[30] Koçak M.Z., Karadağ M., Çelikcan F. (2021). Essential Oil Composition of *Salvia officinalis* and *Rosmarinus officinalis*. J. Agric..

[31] Aebisher D., Cichonski J., Szpyrka E., Masjonis S., Chrzanowski G. (2021). Essential Oils of Seven Lamiaceae Plants and Their Antioxidant Capacity. Molecules.

[32] Grzeszczak J., Wróblewska A., Klimowicz A., Gajewska S., Kucharski Ł., Koren Z.C., Janda-Milczarek K. (2024). Antioxidant Activities of Ethanolic Extracts Obtained from α-Pinene-Containing Plants and Their Use in Cosmetic Emulsions. Antioxidants.

[33] Baccouri B., Rajhi I. (2021). Potential Antioxidant Activity of Terpenes. Terpenes Terpenoids—Recent Advances.

[34] Taibi M., Elbouzidi A., Haddou M., Baraich A., Gharsallaoui A., Mothana R.A., Alqahtani A.M., Asehraou A., Bellaouchi R., Addi M. (2025). Evaluation of the Interaction Between Menthol and Camphor, Major Compounds of Clinopodium Nepeta Essential Oil: Antioxidant, Anti-inflammatory and Anticancer Activities Against Breast Cancer Cell Lines. Chem. Biodivers..

[35] Krishnaiah D., Sarbatly R., Nithyanandam R. (2011). A Review of the Antioxidant Potential of Medicinal Plant Species. Food Bioprod. Process..

[36] Li Y., Huang L., Xu Y., Cheng B., Zhao M. (2024). Antioxidant Mechanism of *Rosmarinus officinalis* Essential Oil Ameliorating Pulmonary Oxidative Stress by Activating NRF2 Signaling Pathway. Res. Sq..

[37] Chrysargyris A., Evangelides E., Tzortzakis N. (2021). Seasonal Variation of Antioxidant Capacity, Phenols, Minerals and Essential Oil Components of Sage, Spearmint and Sideritis Plants Grown at Different Altitudes. Agronomy.

[38] Tekguler B., Koca I., Karadeniz B., Zannou O., Pashazadeh H. (2021). Antioxidant Properties and Monoterpene Composition of 13 Different Pine Resin Samples from Turkey. Commagene J. Biol..

[39] Wojtunik-Kulesza K., Dubiel M., Klimek K. (2025). Metal Ion Reduction, Chelation, and Cytotoxicity of Selected Bicyclic Monoterpenes and Their Binary Mixture. Metabolites.

[40] Göze İ., Vural N., Ercan N. (2016). Characterization of Essential Oil and Antioxidant Activities of Some Species of Salvia in Turkey. Nat. Volatiles Essent. Oils.

[41] Beretta G., Artali R., Facino R.M., Gelmini F. (2011). An Analytical and Theoretical Approach for the Profiling of the Antioxidant Activity of Essential Oils: The Case of *Rosmarinus officinalis* L.. J. Pharm. Biomed. Anal..

[42] Mishra A., Mishra A., Chattopadhyay P. (2012). Assessment of in Vitro Sun Protection Factor of *Calendula officinalis* L. (Asteraceae) Essential Oil Formulation. J. Young Pharm..

[43] Dawidowicz A.L., Olszowy M. (2014). Does Antioxidant Properties of the Main Component of Essential Oil Reflect Its Antioxidant Properties? The Comparison of Antioxidant Properties of Essential Oils and Their Main Components. Nat. Prod. Res..

[44] Bounimi S., Chebli B. (2017). Synergistic Antioxidant Activity of Three Essential Oils of Lamiacea Family from Morocco. J. Environ. Eng. Sci..

[45] Gonzalez-Burgos E., Gomez-Serranillos M.P. (2012). Terpene Compounds in Nature: A Review of Their Potential Antioxidant Activity. Curr. Med. Chem..

[46] Poole K. (2011). Pseudomonas Aeruginosa: Resistance to the Max. Front. Microbiol..

[47] Li X.Z., Nikaido H. (2009). Efflux-Mediated Drug Resistance in Bacteria: An Update. Drugs.

[48] Rouvier F., Brunel J.M., Pagès J.M., Vergalli J. (2025). Efflux-Mediated Resistance in Enterobacteriaceae: Recent Advances and Ongoing Challenges to Inhibit Bacterial Efflux Pumps. Antibiotics.

[49] Lawrence H.A., Palombo E.A. (2009). Activity of Essential Oils against *Bacillus subtilis* Spores. J. Microbiol. Biotechnol..

[50] Nazzaro F., Fratianni F., De Martino L., Coppola R., De Feo V. (2013). Effect of Essential Oils on Pathogenic Bacteria. Pharmaceuticals.

[51] Silva N., Alves S., Gonçalves A., Amaral J.S., Poeta P. (2013). Antimicrobial Activity of Essential Oils from Mediterranean Aromatic Plants against Several Foodborne and Spoilage Bacteria. Food Sci. Technol. Int..

[52] Bowbe K.H., Salah K.B.H., Moumni S., Ashkan M.F., Merghni A. (2023). Anti-Staphylococcal Activities of *Rosmarinus officinalis* and *Myrtus communis* Essential Oils through ROS-Mediated Oxidative Stress. Antibiotics.

[53] Hussain A.I., Anwar F., Hussain Sherazi S.T., Przybylski R. (2008). Chemical Composition, Antioxidant and Antimicrobial Activities of Basil (*Ocimum basilicum*) Essential Oils Depends on Seasonal Variations. Food Chem..

[54] Balouiri M., Sadiki M., Ibnsouda S.K. (2016). Methods for in Vitro Evaluating Antimicrobial Activity: A Review. J. Pharm. Anal..

[55] Hulankova R. (2024). Methods for Determination of Antimicrobial Activity of Essential Oils In Vitro—A Review. Plants.

[56] Yang J., Goksen G., Zhang W. (2023). Rosemary Essential Oil: Chemical and Biological Properties, with Emphasis on Its Delivery Systems for Food Preservation. Food Control.

[57] Kamel D.G., Mansour A.I.A., El-Diin M.A.H.N., Hammam A.R.A., Mehta D., Abdel-Rahman A.M. (2022). Using Rosemary Essential Oil as a Potential Natural Preservative during Stirred-like Yogurt Making. Foods.

[58] Fiume M.M., Bergfeld W.F., Belsito D.V., Hill R.A., Klaassen C.D., Liebler D.C., Marks J.G., Shank R.C., Slaga T.J., Snyder P.W. (2018). Safety Assessment of *Rosmarinus officinalis* (Rosemary)-Derived Ingredients as Used in Cosmetics. Int. J. Toxicol..

[59] Khalifi Taghzouti O., Balouiri M., Ouedrhiri W., Chahad A.E., Romane A. (2016). In Vitro Evaluation of the Antioxidant and Antimicrobial Effects of *Globularia alypum* L. Extracts. J. Mater. Environ. Sci..

[60] Barradas T.N., de Holanda e Silva K.G. (2021). Nanoemulsions of Essential Oils to Improve Solubility, Stability and Permeability: A Review. Environ. Chem. Lett..

[61] Sharmeen J.B., Mahomoodally F.M., Zengin G., Maggi F. (2021). Essential Oils as Natural Sources of Fragrance Compounds for Cosmetics and Cosmeceuticals. Molecules.

[62] Shahrivari S., Alizadeh S., Ghassemi-Golezani K., Aryakia E. (2022). A Comprehensive Study on Essential Oil Compositions, Antioxidant, Anticholinesterase and Antityrosinase Activities of Three Iranian Artemisia Species. Sci. Rep..

[63] Bunse M., Daniels R., Gründemann C., Heilmann J., Kammerer D.R., Keusgen M., Lindequist U., Melzig M.F., Morlock G.E., Schulz H. (2022). Essential Oils as Multicomponent Mixtures and Their Potential for Human Health and Well-Being. Front. Pharmacol..

[64] Morais S.V.d., Mendonça P.G., Vasconcelos C.C., Lopes P.L.A., Garcia J.B.S., Calzerra N.T.M., Queiroz T.M.d., Lima S.T.d.J.R.M., Silva G.E.B., Lopes A.J.O. (2023). Cuminaldehyde Effects in a MIA-Induced Experimental Model Osteoarthritis in Rat Knees. Metabolites.

[65] Mollica F., Gelabert I., Amorati R. (2022). Synergic Antioxidant Effects of the Essential Oil Component γ-Terpinene on High-Temperature Oil Oxidation. ACS Food Sci. Technol..

[66] Bian M., Ma Q.Q., Wu Y., Du H.H., Guo-hua G. (2021). Small Molecule Compounds with Good Anti-Inflammatory Activity Reported in the Literature from 01/2009 to 05/2021: A Review. J. Enzym. Inhib. Med. Chem..

[67] Abderrahim A., Belhamel K., Chalard P., Figuérédo G. (2018). Correlation between Chemical Composition and Antioxidant Activity of the Essential Oils from Leaves and Berries of *Schinus molle* L. Growing in Two Areas of Bejaia (Algeria). J. Food Meas. Charact..

[68] Figueiredo A.C., Barroso J.G., Pedro L.G., Scheffer J.J.C. (2008). Factors Affecting Secondary Metabolite Production in Plants: Volatile Components and Essential Oils. Flavour Fragr. J..

[69] Adams R.P., Thomas P., Rushforth K. (2007). The Leaf Essential Oils of the New Conifer Genus, *Xanthocyparis*: *Xanthocyparis Vietnamensis* and *X*. nootkatensis. J. Essent. Oil Res..

[70] Scherer R., Godoy H.T. (2009). Antioxidant Activity Index (AAI) by the 2,2-Diphenyl-1-Picrylhydrazyl Method. Food Chem..

[71] Re R., Pellegrini N., Proteggente A., Pannala A., Yang M., Rice-Evans C. (1999). Antioxidant Activity Applying an Improved ABTS Radical Cation Decolorization Assay. Free Radic. Biol. Med..

[72] Oyaizu M. (1986). Antioxidative Activities of Browning Reaction Prepared from Glucosamine. Jpn. J. Nutr..

[73] Kunchandy E., Rao M.N.A. (1990). Oxygen Radical Scavenging Activity of Curcumin. Int. J. Pharm..

[74] Özyürek M., Güngör N., Baki S., Güçlü K., Apak R. (2012). Development of a Silver Nanoparticle-Based Method for the Antioxidant Capacity Measurement of Polyphenols. Anal. Chem..

[75] Karthik K., Thangaswamy V. (2013). Evaluation of Implant Success: A Review of Past and Present Concepts. J. Pharm. Bioallied Sci..

[76] El Atki Y., Aouam I., El Kamari F., Taroq A., Nayme K., Timinouni M., Lyoussi B., Abdellaoui A. (2019). Antibacterial Activity of Cinnamon Essential Oils and Their Synergistic Potential with Antibiotics. J. Adv. Pharm. Technol. Res..

[77] Hans V.M., Grover H.S., Deswal H., Agarwal P. (2016). Antimicrobial Efficacy of Various Essential Oils at Varying Concentrations against Periopathogen *Porphyromonas gingivalis*. J. Clin. Diagn. Res..

[78] Hurtado R., Peltroche N., Mauricio F., Gallo W., Alvítez-Temoche D., Vilchez L., Mayta-Tovalino F. (2020). Antifungal Efficacy of Four Different Concentrations of the Essential Oil of *Cinnamomum zeylanicum* (Canela) against *Candida albicans*: An in Vitro Study. J. Int. Soc. Prev. Community Dent..

[79] Messaoudi M., Rebiai A., Sawicka B., Atanassova M., Ouakouak H., Larkem I., Egbuna C., Awuchi C.G., Boubekeur S., Ferhat M.A. (2022). Effect of Extraction Methods on Polyphenols, Flavonoids, Mineral Elements, and Biological Activities of Essential Oil and Extracts of *Mentha pulegium* L.. Molecules.

[80] Prabuseenivasan S., Jayakumar M., Ignacimuthu S. (2006). In Vitro Antibacterial Activity of Some Plant Essential Oils. BMC Complement. Altern. Med..

